# Low-resource MobileBERT for emotion recognition in imbalanced text datasets mitigating challenges with limited resources

**DOI:** 10.1371/journal.pone.0312867

**Published:** 2025-01-24

**Authors:** Muhammad Hussain, Caikou Chen, Sami S. Albouq, Khlood Shinan, Fatmah Alanazi, Muhammad Waseem Iqbal, M. Usman Ashraf

**Affiliations:** 1 College of Information Engineering Yangzhou University, Yangzhou, PR China; 2 Faculty of Computer and Information Systems, Islamic University of Madinah, Madinah, Saudi Arabia; 3 Department of Computers, College of Engineering and Computers in Al-Lith, Umm Al-Qura University, Makkah, Saudi Arabia; 4 Computer Science Department, College of Computer and Information Sciences, Imam Muhammad Bin Saud University, Riyadh, Saudi Arabia; 5 Department of Software Engineering, Superior University Lahore, Lahore, Pakistan; 6 Department of Computer Science, GC Women University Sialkot, Sialkot, Pakistan; Oakland University, UNITED STATES OF AMERICA

## Abstract

Modern dialogue systems rely on emotion recognition in conversation (ERC) as a core element enabling empathetic and human-like interactions. However, the weak correlation between emotions and semantics poses significant challenges to emotion recognition in dialogue. Semantically similar utterances can express different types of emotions, depending on the context or speaker. In order to tackle this challenge, our paper proposes a novel loss called Focal Weighted Loss (FWL) with adversarial training and the compact language model MobileBERT. Our proposed loss function handles the problem of imbalanced emotion classification through Focal Weighted Loss and adversarial training and does not require large batch sizes or more computational resources. Our approach has been employed on four text emotion recognition benchmark datasets, MELD, EmoryNLP, DailyDialog and IEMOCAP demonstrating competitive performance. Extensive experiments on these benchmark datasets validate the effectiveness of our proposed FWL with adversarial training. This enables more human-like interactions on digital platforms. Our approach shows its potential to deliver competitive performance under limited resource constraints, comparable to large language models.

## 1 Introduction

Emotions are a fundamental aspect of human communication and decision-making. In the expanding universe of online communication, social media, and the corporate world, the ability to accurately identify emotions within text has become a critical area of interest, bridging the gap between human nuances and digital interpretation. However, capturing the contextual semantics of personal experiences described in one’s utterance is challenging. Understanding and recognising these emotions can significantly enhance chatbot responsiveness, refine healthcare communication, and improve the analysis of social media [1] content for emotion, sentiment, and opinion mining. This, in turn, can lead to increased customer satisfaction and higher conversion rates. Emotion recognition during conversation has attracted significant attention from both academia and the corporate world [2, 3] due to the day by day growing usage and popularity of online social media and other social networking platforms such as Facebook, Twitter, YouTube, and other popular social media platforms [4]. Emotions expressed during conversation are of a dynamic nature and can be influenced by a variety of factors [5] including speaker dependence and the surrounding conversational environment [6].

However, the text emotion recognition task faces several challenges. Depending on the emotional context, similar emotion utterances may exhibit entirely different emotional attributes. Simultaneously, distinguishing conversation texts that contain similar emotional attributes is also extremely difficult [7]. As a result, significant implicit efforts have been made to construct distinctive utterance representations from two lines, consisting of model creation and representation learning. As a representation from the previous line [4] create and design recurrent models to track dialogue emotion recognition history for the classification task. Representation learning approaches mostly use supervised contrastive learning [8] for learning emotion recognition utterance representations [5], a prototypical contrastive learning technique to address the issue of class imbalance problems and achieve best performance as compared to the previous work, but still, they are struggling to improve the performance of minority classes. The findings demonstrate that similar emotions, such as excited and happy, are frequently misclassified as each other. Supervised contrastive learning is still facing difficulty with effectively differentiating similar text emotions. On the other hand, multimodal emotion recognition, while facial and speech emotion recognition methods have advanced significantly, text-based emotion recognition still demands further exploration and research [9]. Text emotion recognition is particularly challenging due to the way emotions are expressed and how the meanings of utterances vary based on the particular topic discussed, as well as the implicit knowledge shared between participants. Emotional dialogues around specific topics carry certain language patterns, affecting not only the utterance’s meaning but also the particular emotions conveyed by specific expressions.

Online text emotion recognition predicts events, opinions, and attitudes from social media and digital platforms, providing valuable insights into public emotions and preferences [10, 11]. Emotion recognition enables proactive responses to potential situations by analysing conversations and identifying emotions in real-time. Unlike sentiment analysis, which focuses on determining the sentiment of a text as a whole, emotion recognition aims to accurately identify the emotions expressed by each individual in a conversation. Accurately recognising the conversion is a difficult task because it demands a deep understanding of contextual cues, such as tone, nuance, and subtlety, to capture the emotions expressed by each speaker. There are various significant applications of emotion recognition, including social media monitoring, customer service, and political campaign analysis. For instance, commercial and government entities can gain valuable insights into public opinion and sentiment on various topics by analysing text data from social media platforms, online forums, and customer feedback. This process involves extracting and analysing emotions, sentiments, and public opinions to understand public attitudes towards different issues [12, 13]. By leveraging emotion recognition and sentiment analysis, businesses can gain a deeper understanding of their customer behaviours and attitudes to make better decisions in marketing, product development, and customer service.

**Fig 1:**
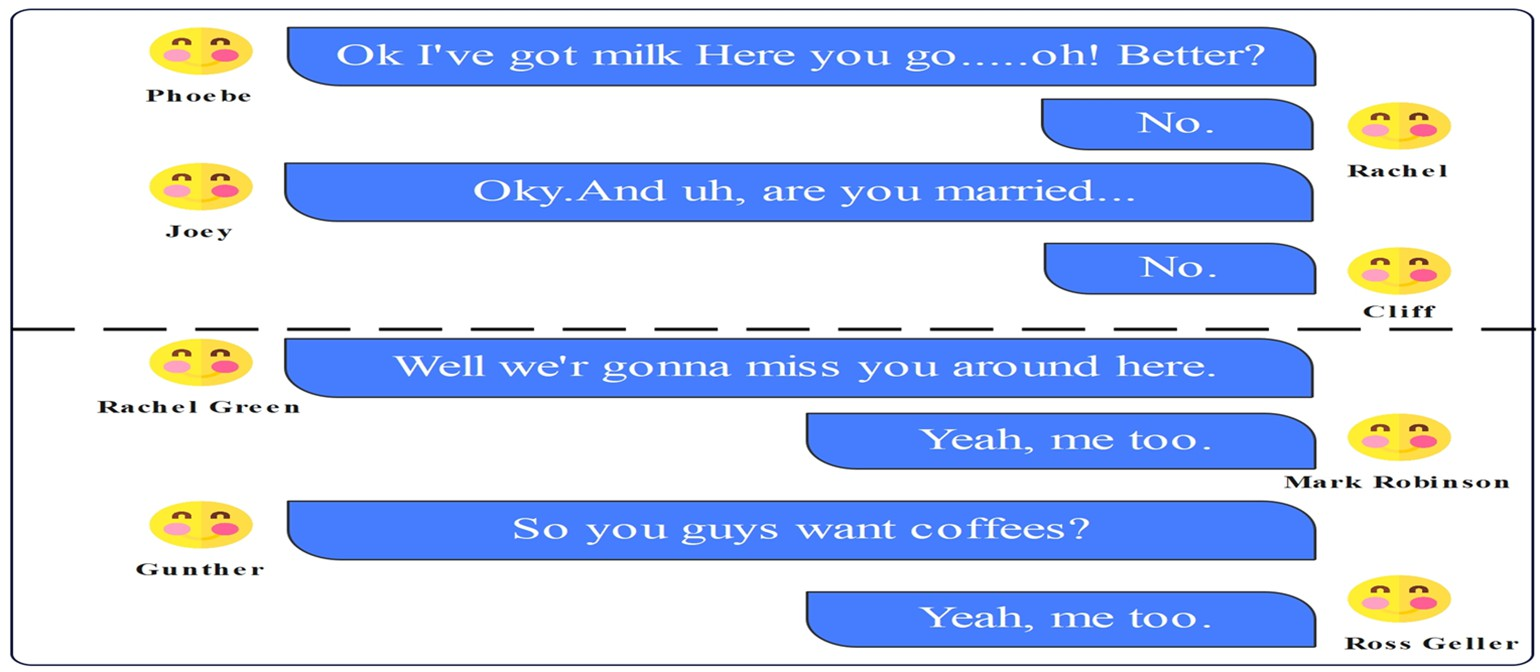
Emotion recognition in conversation. No. and Yeah, me too.

However, emotion recognition models deep learning and transformers are computationally complex models that are computationally expensive to implement on low-resource devices like mobile devices. Although deep learning and transformer models are reported to have interesting findings, these approaches may not be feasible for computation-constrained devices. In this paper, we propose a loss function combined with a low-computability MobileBERT model that is capable of working with low computational resources without introducing unnecessary complexity. Emotion recognition in conversation. "No." and “Yeah, me too.” (Fig 1) can convey different emotions in different contexts.

To the extent of our knowledge, we are the first to use Focal Weighted Loss with adversarial training for the emotion recognition task, significantly enhancing the robustness and sensitivity of MobileBERT in the recognition of nuanced emotional expressions.

- Novel Integration of Focal Weighted Loss with Adversarial Training: We implement the combination of Focal Weighted Loss with adversarial training for emotion recognition, leading to improved robustness and accuracy in MobileBERT.
- Enhanced Emotion Recognition with Limited Resources: We demonstrated the effectiveness of our approach in achieving state-of-the-art results on four benchmark datasets (MELD, EmoryNLP, DailyDialog, and IEMOCAP) with limited computational resources.
- Improved MobileBERT Performance: Our technique significantly enhanced MobileBERT’s performance on emotion recognition tasks, achieving accuracies of 63% on MELD, 43% on EmoryNLP, 62% on DailyDialog, and 63% on IEMOCAP.
- Effective Management of Class Imbalance: We leveraged Focal Weighted Loss to mitigate class imbalance issues, ensuring that MobileBERT learnt to recognise emotions from minority classes more effectively.
- Adversarial Training for Robustness: We implemented adversarial training to improve MobileBERT’s ability to handle perturbations and nuances in emotional expressions, leading to more reliable emotion recognition.

## 2 Related work

### 2.1 Emotion recognition

Emotion recognition is not a single field but an interdisciplinary field of research, with contributions from different fields such as computer vision, natural language processing, and psychological cognitive science. There are various applications of emotion recognition in various different domains like opinion mining, social media, medical and health care chat bots, human-computer interaction, and customer service. Research has explored emotion recognition in various forms of text data, including movie dialogues, instant messaging services, tweets [14], public comments, and news headlines [15]. However, emotion recognition in conversational scenes presents a unique challenge, differing significantly from analysing shorter texts like tweets or social media posts. Unlike context-independent shorter texts, where emotions are solely derived from the words used, whereas conversational texts are context-dependent, with the conversation history and speaker turns influencing the emotions expressed by other speakers [16]. Advancements in emotion recognition rely on comprehensive datasets and addressing specific research challenges. Multimodal emotion recognition, combining text, speech, and facial expressions, enhances accuracy and reliability [17]. These fusions highlight emotion recognition in conversation and its dynamic role in developing empathetic communication and human-machine interactions.

Most existing approaches to emotion recognition in conversion are graph-based approaches, sequence-based approaches, knowledge-enhanced based approaches, and transformer-based approaches. Graph-based approach methods DialogGCN [18], RGAT [19] Construct a graph using the utterance nodes. Represents speakers and utterances as nodes and constructs a unified graph where ConGCN [20] uses the whole emotion recognition in the conversion dataset. Utilises a directed acyclic graph, DAG-ERC [21], to represent the intrinsic structure within a conversation. However, knowledge enhanced base models utilise external knowledge [22–24]. The sequence-based approaches determine contextual information as sequences of utterances use the gated recurrent unit for capturing context information ICON [25], HiGRU [26]. Uses recurrent neural network methods to represent conversation dynamics DialogRNN [4]. The multi-turn reasoning modules [27] analyse the ERC problem from a cognitive perspective. Use context and the speaker’s memory using pre-trained language models CoMPM [28]. Usually, they utilise external knowledge from ATIMOC [29]. The capabilities of language models include CoMPM [28], which leverages the language model’s ability to learn and track contextual information. A transformer based approach supposes that each utterance is regarded as an independent sentence, regardless of its context dependence and speaker information. The problem can be transformed into a simple sentence classification so that pre-trained models [30–32] can be used directly for fine-tuning. Adopts BERT [33] to extract features from utterances, followed by transformer structure for modelling context. Speaker dependence, considering the auxiliary task of judging whether two utterances are from the same speaker, is used to model the speaker information. COSMIC [24] exploits RoBERTa as the feature extractor for each utterance and models the contextual dependency with RNN. In addition, the common knowledge transformer COMET [34] is incorporated to introduce world knowledge. However, supervised contrastive focus on ERC and apply supervised contrastive loss as an additional optimisation goal. To make full use of label information [35], extend it on self-supervised training so that samples belonging to the same label are gathered in the embedding space while pushing samples of different categories apart.

### 2.2 Genesis (evolution) pre-training transformer-based language models

The seminal work on pre-training introduced the transformer architecture for language comprehension [36] using a two-stage training approach self-supervised pre-training on a generic text corpus and fine-tune training on specific application data [37]. Initially, bi-directional RNNs were pre-trained for contextualised token representations. However, RNN-based models face limitations in long-term modelling and scaling due to recurrent connections. In contrast, transformer-based models have revolutionised the field through parallel processing and deep model development, achieving state-of-the-art results in various downstream NLP tasks BERT [30], ELECTRA [38], and GPT [39] have been implemented and have achieved state-of-the-art performance in several downstream natural language processing tasks. Various studies, such as transfer learning ERC [40] utterance-level dialogue understanding [41], and contextualised emotion tagging [42], despite their potential, transformer-based models have not yet fully addressed emotions. Pre-trained language models like BERT [43] have significantly advanced NLP, including emotion recognition sentiment analysis text summarization and name entity recognition, but computational requirements of large language model computational resources and required memory limit their deployment in resource-constrained environments. To address this issue, we have utilised the low-resource MobileBERT model for text emotion recognition, which offers a more efficient solution for resource-constrained settings. By leveraging the efficient and powerful MobileBERT architecture, our model integrates Focal Weighted Loss and adversarial training to achieve robust and optimal emotion recognition in conversations. By combining these techniques, we create a powerful fusion that enhances the model’s ability to capture subtle emotional cues and generalise well to unseen data, ultimately improving the reliability and performance of ERC systems.

## 3 Methodology

### 3.1 Problem statement

Given a collection of speakers S, an array of emotion labels E, and a specific conversation C, the goal of our research is to determine the emotional state of the speakers inside the conversation. There is a predetermined order to the conversational turns, which are [(*s*_1_, *u*_1_), (*s*_2_, *u*_2_), (*s*_3_, *u*_3_)……… (*s*_*n*_, *u*_*n*_)], where *s*_*i*_ denotes a speaker from the set *S* and *u*_*i*_ is the utterance of speaker *s*_*i*_ during the *i*^*th*^ turn. Our research is focused on the dynamics of emotion recognition in conversation in real time. In this case, the model is set up to predict the emotion label with Focal Weighted Loss to enhance the performance of emotion recognition. We propose the Focal Weighted Loss function according to the prediction certainty in order to fine-tune the model to better focus on emotionally difficult minority classes. It predicts the emotion label *y*^*t*^ for the current turn t using contextual and sequential data from previous turns [(*s*_1_, *u*_1_), (*s*_2_, *u*_2_), (*s*_3_, *u*_3_) (*s*_*t*_, *u*_*t*_)]. In addition, we take advantage of adversarial training to improve the model’s capability to identify intricate expressions and enhance its ability to adjust to challenges.

**Algorithm:** Training Process for emotion recognition with Focal Weighted Loss and adversarial training

 **Dtrain**: the training dataset

 **R**: the total number of epochs

 **M**: MobileBERT for sequence classification

 **E**: emotion labels

 **α, γ**: parameters for focal loss, adjusted dynamically

 **ε**: perturbation magnitude for adversarial training

**Outputs:**

 **M’**: the optimally trained model

 **C**_**j**_: category centers for each label

**Steps:**

**1. Initialization:**

 Initialize model M with MobileBERT.

 Initialize parameters α and γ.

**2. Training Loop:**

  for *k* = 0 to *R* do:

  for each batch (*X*,*y*) in *Dtrain*

 **1. Forward Pass:**

  Compute logits: logits = *M*(*X*)

  Calculate probabilities using softmax on logits *p*_*t*_

 **2. Focal Weighted Loss Calculation**:

  *FL* (*p*_*t*_) = − *α*_*t*_(*y*) ⋅ (1−*p*_*t*_)^*γ*^ ⋅ log (*p*_*t*_)

  *α*_*t*_ (*y*) is dynamically adjusted based on the misclassification rate of class *y* or its representation in the batch.

  *γ* is adjusted based on the overall performance or specific training epoch.

  Combine losses to form the batch loss:

L=mean(FL)

 **3. Adversarial Training:**

  Generate adversarial examples:

$$X_{adv}=X+\epsilon \cdot \text{sign}\;(\nabla_{X}L)$$

  Compute logits for adversarial examples

$${\text{logits}}_{adv}=M(X_{adv})$$

  where *M* represents the model, and *X*_*adv*_ are the adversarial examples

  Compute the loss for adversarial examples:

$$L_{adv}=\text{Loss}\;(Y,\;{\text{logits}}_{adv})$$

  where *Y* are the true labels

 **4. Total Loss Calculation**:

  Compute the total loss by combining the original and adversarial losses:

$$L_{total}=L_{orig}+L_{adv}$$

  where *L*_*orig*_ is the loss computed using the original data.

 **5. Backpropagation and Optimization:**

   *L*_*total*_. *backward* ()

  Update model parameters using an optimizer:

   optimizer.step ()

   optimizer.zero_grad ()

  End of batch processing.

  End of epoch processing.

**3. Return:**

 Return the optimally trained model *M*′ and category centers *C*_*j*_.

Dynamic Alpha Adjustment: Adjust *α* based on ongoing training insights. For underrepresented classes, or those with higher misclassification rates, increase *α* to give more weight to their errors.

Gamma Tuning: Adapt *γ* to make the model more sensitive to misclassified examples as training progresses, potentially increasing *γ* to focus more on harder cases

### 3.2 Low resource MobileBERT with focal weighted loss

The MobileBERT model is a compact and efficient thin version of the large language model BERT_LARGE [44] designed for computationally constrained mobile devices with optimised performance and processing speed in real-time applications. The MobileBERT model is fine-tuned for a wide range of language processing tasks while offering good performance. MobileBERT is 4.3× smaller and 5.5× faster than BERT_BASE_ Model. We boost the capabilities of the MobileBERT model by integrating Focal Weighted Loss, a modified loss function that builds upon traditional cross-entropy loss [45]. Unlike standard cross-entropy loss, Focal Weighted Loss incorporates a modulating factor in which the loss contribution from each class is dynamically adjusted based on its classification difficulty. This strategic approach allows the model to focus on difficult and minority cases and effectively address class imbalances, particularly in datasets with skewed emotional expression distributions. By integrating Focal Weighted Loss with the MobileBERT model, we significantly improve the model’s performance to recognise and distinguish between various emotional states from text. The model can capture emotional nuances well, understand context more effectively, and gain a more comprehensive understanding of emotional nature. This leads to overall enhanced performance and more accurate recognition of emotional states.

$$FL\left(p_{t}\right)=-\alpha_{t}{\left(1-p_{t}\right)}^{\gamma }\;\text{log}\left(p_{t}\right)+\lambda CWL$$ (1)

Where:

*p*_*t*_ is the model estimated probability for class *t*. *α*_*t*_ adjusts the importance of each class, addressing class imbalance. (1 − *p*_*t*_)^*γ*^ is the focusing term reduces the loss contribution from easy examples allowing the model to focus on harder misclassified examples. log(*p*_*t*_) log loss penalises incorrect predictions. *λCWL* class weighted loss adds additional class-weighted penalties to further address class imbalance. By integrating these elements, the FWL function effectively balances the attention given to minority classes and hard-to-classify examples, resulting in improved performance and robustness in low-resource text emotion recognition tasks.

### 3.3 Contextual adversarial training (CAT)

Employ contextual adversarial training [46, 47] to enhance the robustness of our low-resource MobileBERT model against adversarial attacks. Unlike traditional adversarial training methods that add perturbations to context-free layers, the contextual adversarial training (CAT) strategy applies adversarial perturbations to the context-aware network structure in a multi-channel way. This technique allows us to obtain diverse context features and improve the model’s robustness to contextual perturbations.

Let (*u*, *y*) denote the mini-batch input sampled from distribution *D*, where *u* represents the input features and *y* the corresponding labels. The context-aware model outputs probabilities *p*(*y*|*u*; *θ*), where *θ* denotes the model parameters. During each training step, we introduce contextual adversarial perturbations *r*_*c-adv*_, computed against the current model parameters $\hat{\theta }$, and incorporate them into the context-aware hidden layers. The perturbations are generated using the following linear approximation under an *L*_*q*_ norm constraint with radius *ϵ*:

$$r_{c-adv}=\epsilon .sign\left(\nabla_{u}\;J\left(\theta ,u,y\right)\right)$$ (2)

Where ∇_*u*_J(*θ*, *u*, *y*) gradient of the loss function J with respect to the input *u*, which indicates how the loss changes with small changes in the input.

### 3.4 Adversarial training procedure

#### Loss function

Implement the **Focal Weighted Loss,** which combines weighted categorical cross-entropy and focal loss to handle class imbalance:

$$Loss=\left(1-focal_{weight}\right).CE\left(p,y\right)+focal_{weight}.FL\left(p,y\right)$$ (3)

Here, *p* are the model predictions, *CE*(*p*, *y*) cross-entropy loss, which measures the difference between the predicted probabilities *p* and the true labels *y*. It is effective for general classification tasks. whereas *FL*(*p*, *y*) focal loss, which increases the importance of correcting misclassified examples by down-weighting easy examples and focusing on hard-to-classify ones. and focal weight is a weighting factor that determines the contribution of the focal loss in the overall loss function. It ranges from 0 to 1. This loss is particularly beneficial in tasks with class imbalance, such as low-resource text emotion recognition.

### 3.5 Adversarial perturbation generation (FGSM attack)

Compute the gradient of the loss concerning the input embeddings. Adjust the embeddings by a small perturbation *ϵ* epsilon in the direction of the gradient sign.


$$r_{c-adv}\;=\epsilon .sign\left(\nabla_{input\_embeds}Loss\right)$$ (4)

## 4 Experiments

### 4.1 Model setting

**Fig 2:**
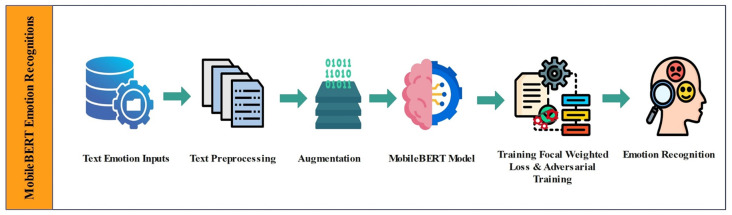
MobileBERT emotion recognition.

We implement our proposed loss function on the MobileBERT model using the PyTorch framework for text emotion recognition tasks on the MELD, EmoryNLP, DailyDialog and IEMOCAP datasets. We use a pre-trained MobileBERT model to extract word embeddings from text transcription. We fine-tune the pre-trained MobileBERT model on each dataset using the Adam optimizer with a learning rate of 2e-5 and train for 4, 8, 10, and 12 epochs with a batch size of 32 and 64. We utilise the Focal Weighted Loss and adversarial training functions to address class imbalance issues in the datasets. Our model achieves performance comparable to large language models while requiring fewer resources, achieving robust and near-optimal results on four text emotion recognition datasets. Below (Fig 2) is the representation of our model and how it’s working.

### 4.2 Model training

We trained the MobileBERT model on a Windows 10 PC with 32 GB of RAM, an RTX 3060 6 GB dedicated GPU, and a Core i7 2.60 GHz processor using Python 3.8.2. We employed the Adam optimizer with a learning rate of 2e-5 and trained the model for 12 epochs with a batch size of 64. We utilised the pre-trained MobileBERT model and fine-tuned it on our datasets, MELD, EmoryNLP, DailyDialog and IEMOCAP.

### 4.3 Datasets

**Fig 3:**
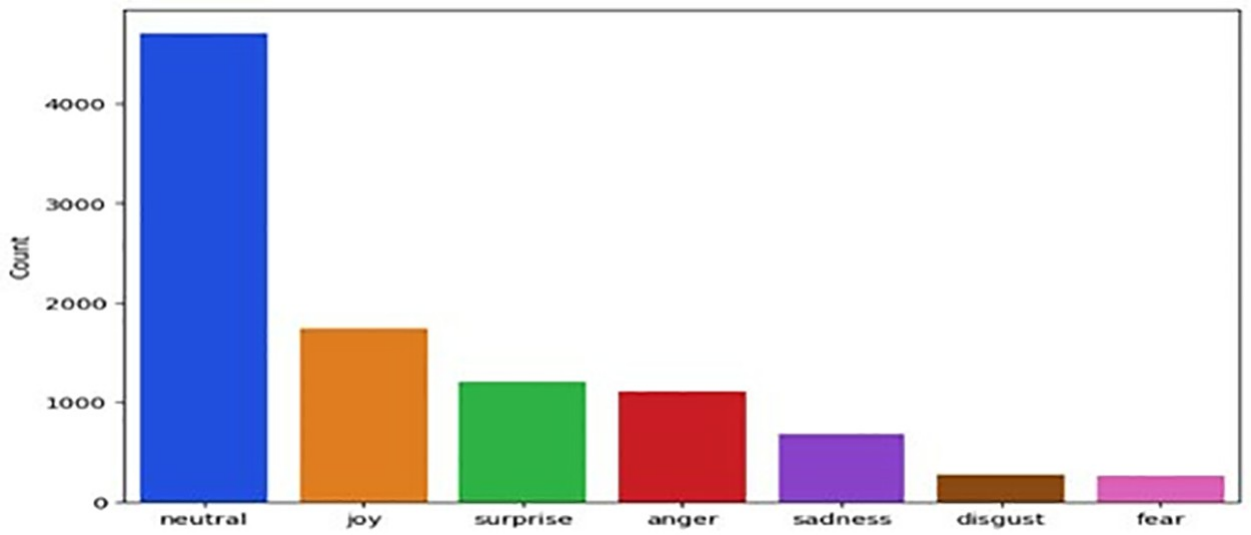
Emotion distribution of MELD.

**MELD** [48] A multi-party conversation corpus was collected from the TV show Friends. Which contains around 1400 conversations and 13000 utterances. Several speakers engaged in the dialogue. Each utterance in a dialogue has been labelled by one of these seven emotions (Fig 3), represent the emotions classes Neutral, joy, surprise, anger, sadness, disgust, and fear. We have used text-based emotion recognition.

**Fig 4:**
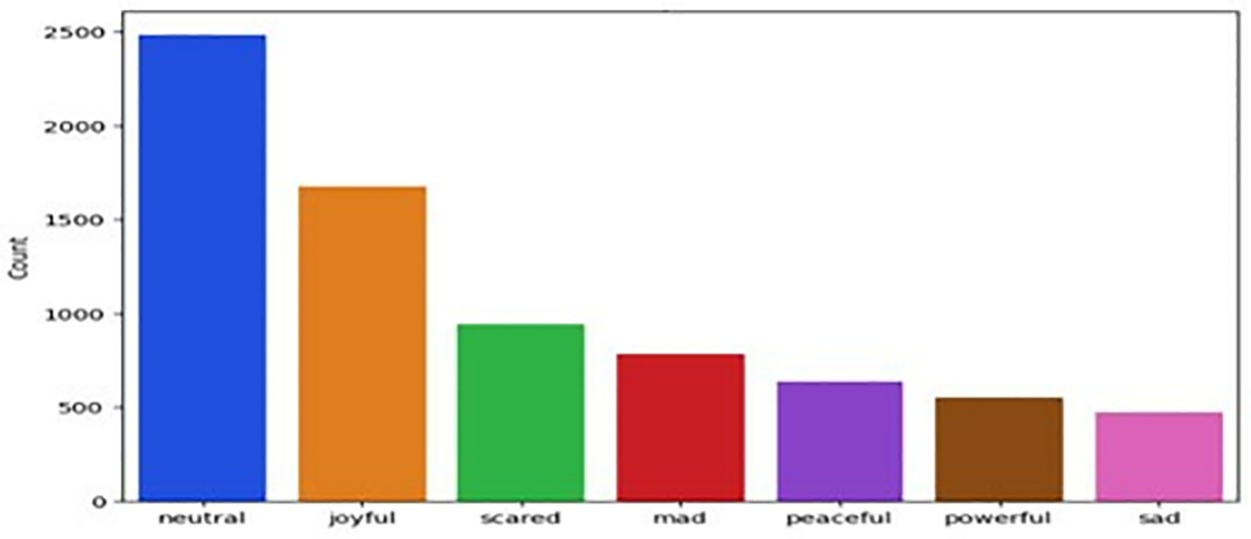
Emotion distribution of EmoryNLP.

**EmoryNLP** [49] A multiparty conversation corpus was collected from friends but varies from MELD in the choice of scenes and emotion labels. This dataset comprises 97 episodes, 897 scenes, and 12,606 utterances, where each utterance is annotated with one of the emotion labels (Fig 4), The emotion class labels are including neutral, joyful, scared, mad, peaceful, powerful, and sad.

**Fig 5:**
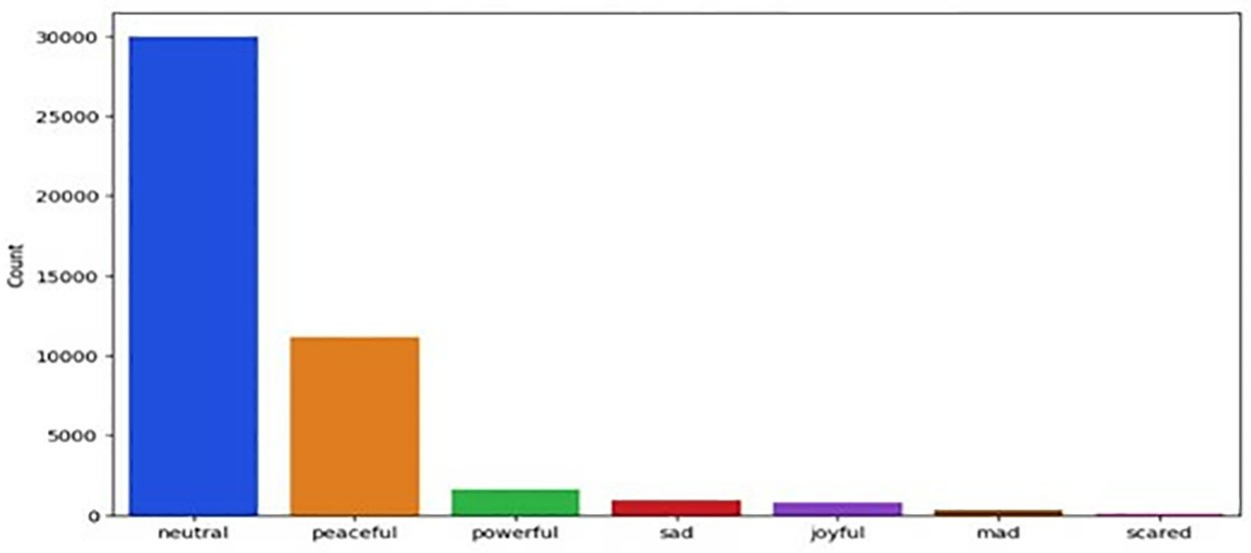
Emotion Distribution of DailyDialog.

**DailyDialog** [50] The DailyDialog dataset corpus is a human-written multiturn dynamic dialogue dataset, reflecting our daily lives and consists of communications from English learners. covering various topics about our daily lives. Each utterance is annotated with an emotion label (Fig 5) from 7 classes: neutral, peaceful, powerful, sad, joyful, mad, and scared.

**Fig 6:**
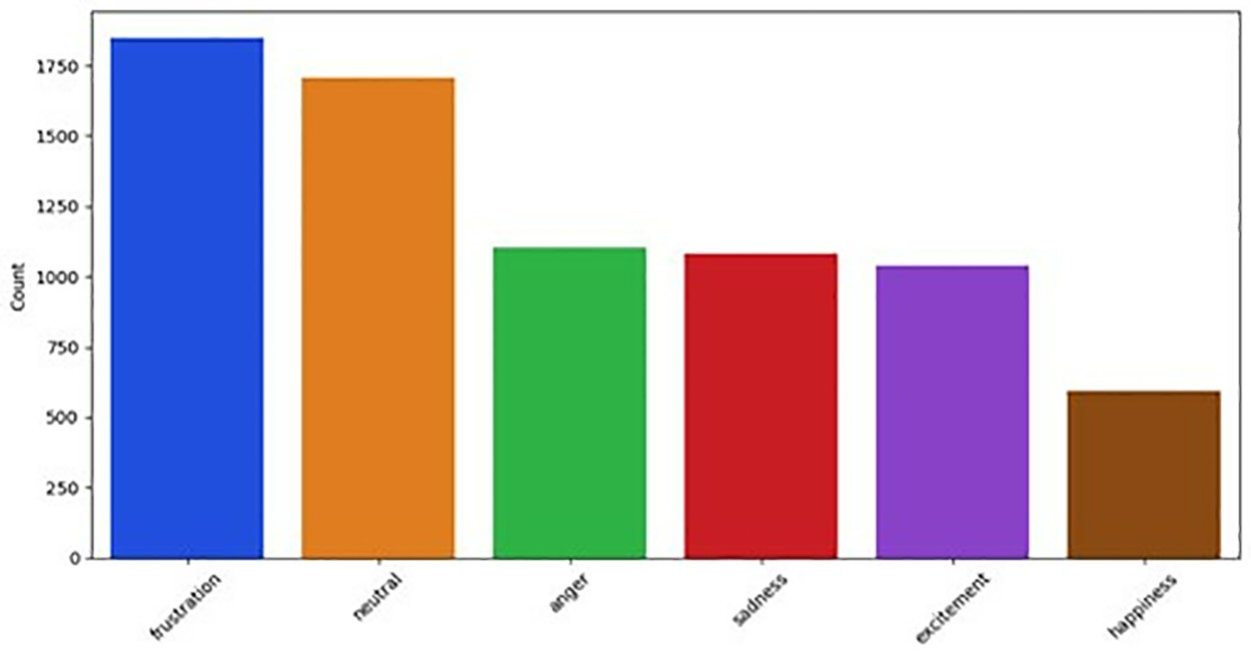
Emotion distribution of IEMOCAP.

**IEMOCAP** [51] The interactive emotional dynamic motion capture IEMOCAP is a dataset that consists of 151 recorded video dialogues, two speakers per session, for a total of 302 videos across the dataset. There are a total of ten different unique speakers. Each dialogue utterance is annotated with one of the following (Fig 6) emotion labels from the six below emotions frustrated, neutral, anger, sadness, excitement, and happiness.

### 4.4 Evaluation metrics

We observe class imbalances in the four benchmark datasets: Table 1 MELD, EmoryNLP, DailyDialog, and IEMOCAP. To address this, we adopt appropriate evaluation metrics Table 2 for each dataset. Specifically, we use the weighted F1 score for MELD, EmoryNLP, and IEMOCAP, consistent with previous research, to account for class imbalance. For the DailyDialog emotion dataset, which does not include neutral classes, we employ the Micro F1 score to evaluate our model’s performance. This enables a fair assessment of our model’s performance and facilitates comparison with previous works.

**Table 1:** Statistics of the MELD, EmoryNLP, DailyDialog, and IEMOCAP.

Dataset	No. Dials	Train	Dev	Test	No.Uttrs	Train	Dev	Test	No.CLS	Evolution Metrics
MELD	1,432	1,038	114	280	13,708	9,989	1,109	2,610	7	Weighted avg F1
EmoryNLP	827	659	89	79	9,489	7,551	954	984	7	Weighted avg F1
DailyDialog	13118	11118	1000	1000	102979	87170	8069	7740	7	Macro F1 & Micro F1
IEMOCAP	151	100	20	31	7,333	4810	1000	1523	6	Weighted avg F1

**Table 2:** Performance comparisons of models.

Model	MELD	EmoryNLP	DailyDialog	IEMOCAP	Ref
DialogRNN+Roberta	63.61	37.44	57.32	64.76	[4]
DialogGCN+Roberta	63.02	38.10	57.52	64.18	[18]
RGAT+Roberta	62.80	37.89	59.02	66.36	[19]
KET	58.18	34.73	53.37	59.56	[52]
DialogXL	62.41	34.73	54.93	65.94	[53]
DAGNN	63.12	37.98	58.36	64.61	[21]
COSMIC	64.28	37.10	56.16	63.05	[24]
DAG-ERC	63.65	39.02	59.33	68.03	[21]
CoMPM	66.52	38.93	60.34	69.46	[28]
T-GCN	65.36	39.24	61.91	-	[54]
TRMSM-Att	62.36	-	-	65.74	[55]
IEmoNet(BE)-A	-	-	-	71.4	[56]
MobileBERT-FWL+AT(Ours)	63	43	62	63	

## 5 Results and discussion

We evaluate the proposed technique against state-of-the-art text-based emotion recognition approaches in conversation, with results presented in Table 3 By integrating our MobileBERT model with Focal Weighted Loss and adversarial training, we achieve optimal performance, outperforming existing methods and demonstrating the effectiveness of our approach, particularly when combined with the efficient MobileBERT model.

**Table 3:** FWL+AT is Focal Weighted Loss with adversarial training.

	MELD	EmoryNLP	DailyDialog	IEMOCAP
FWL+AT	63	43	62	63

### 5.1 Ablation study

To comprehensively evaluate the effectiveness of Focal Weighted Loss (FWL) and adversarial training (AT) on our low-resource MobileBERT model for text emotion recognition, we conducted a thorough ablation analysis. We fine-tuned the pre-trained MobileBERT-based encoder on the MELD, EmoryNLP, DailyDialog and IEMOCAP datasets.

We conduct ablation studies to evaluate our key contribution to MobileBERT with Focal Weighted Loss and adversarial training. When removing the proposed (FWL) and adversarial training loss and replacing it with a simple focal loss, we obtain inferior performance in terms of emotion recognition.

### 5.2 Combining focal weighted loss and adversarial training

By integrating Focal Weighted Loss with adversarial training, we found a powerful combination that effectively utilised the strengths of both approaches. Our results demonstrate that our innovative combination of Focal Weighted Loss with adversarial training delivers the best performance across all four benchmark text emotion recognition datasets, with a low-resource MobileBERT model on imbalanced text emotion recognition tasks.

Our approach lies in its complementary strengths. Focal Weighted Loss effectively addresses class imbalance issues, while adversarial training enhances robustness against adversarial attacks. By integrating these two strategies, we achieve the best overall performance, showcasing the vast potential of this approach for real-world applications.

### 5.3 Hyperparameter tuning

**Fig 7:**
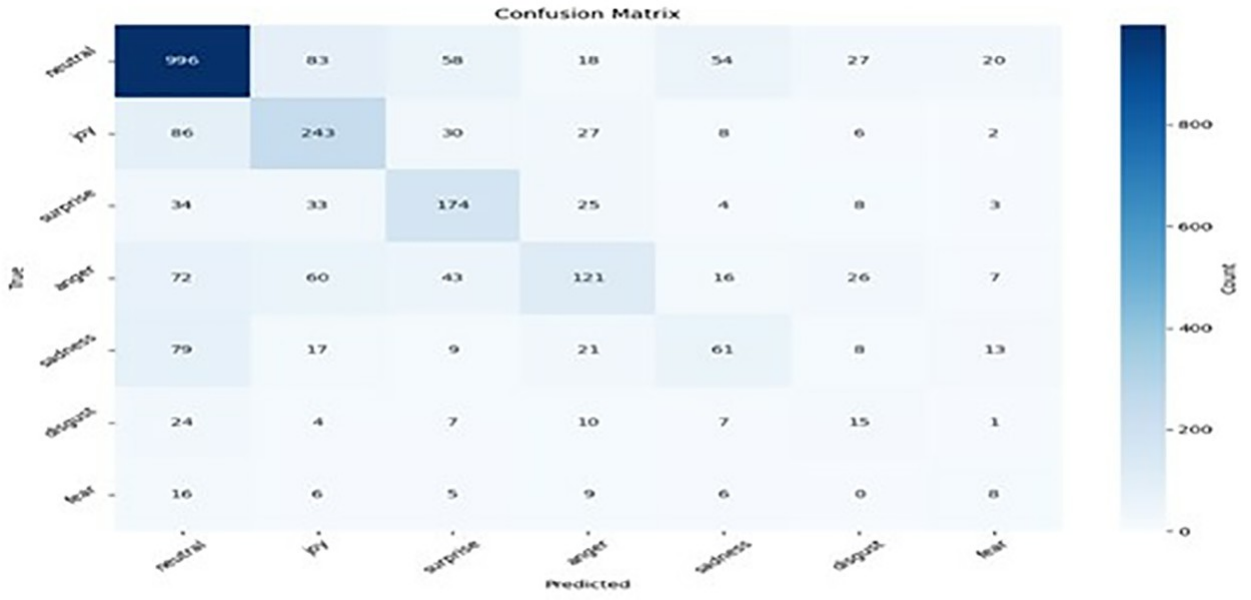
Meld confusion matrix.

**Fig 8:**
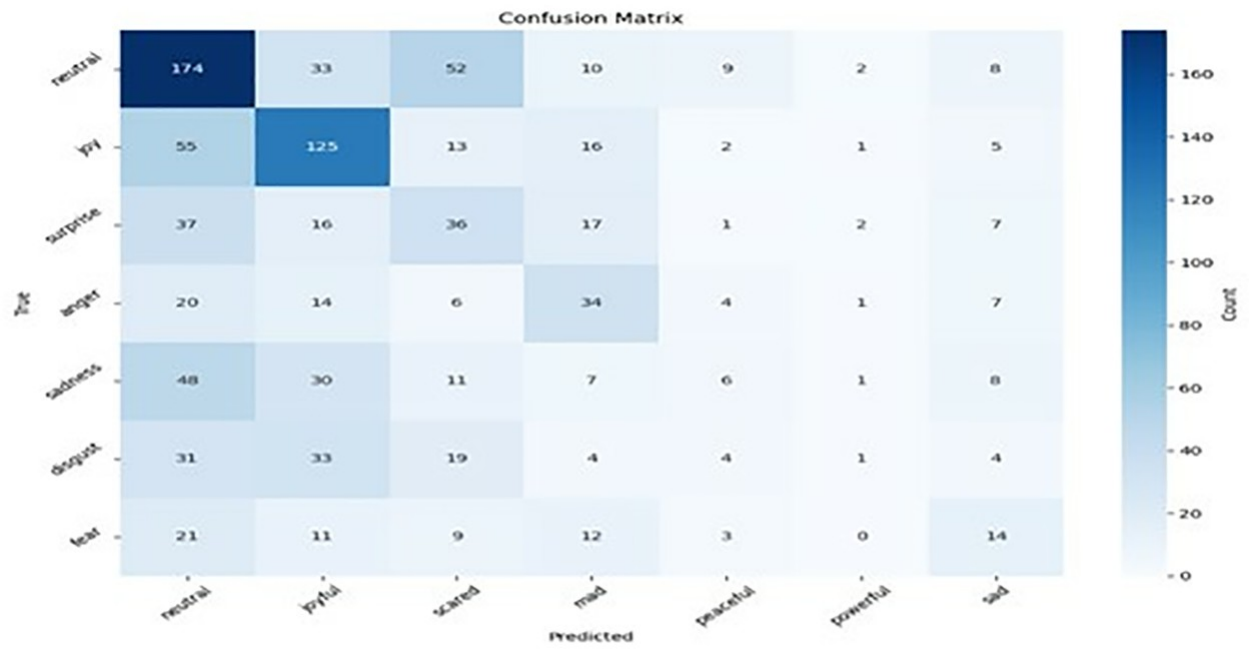
EmoryNLP confusion matrix.

**Fig 9:**
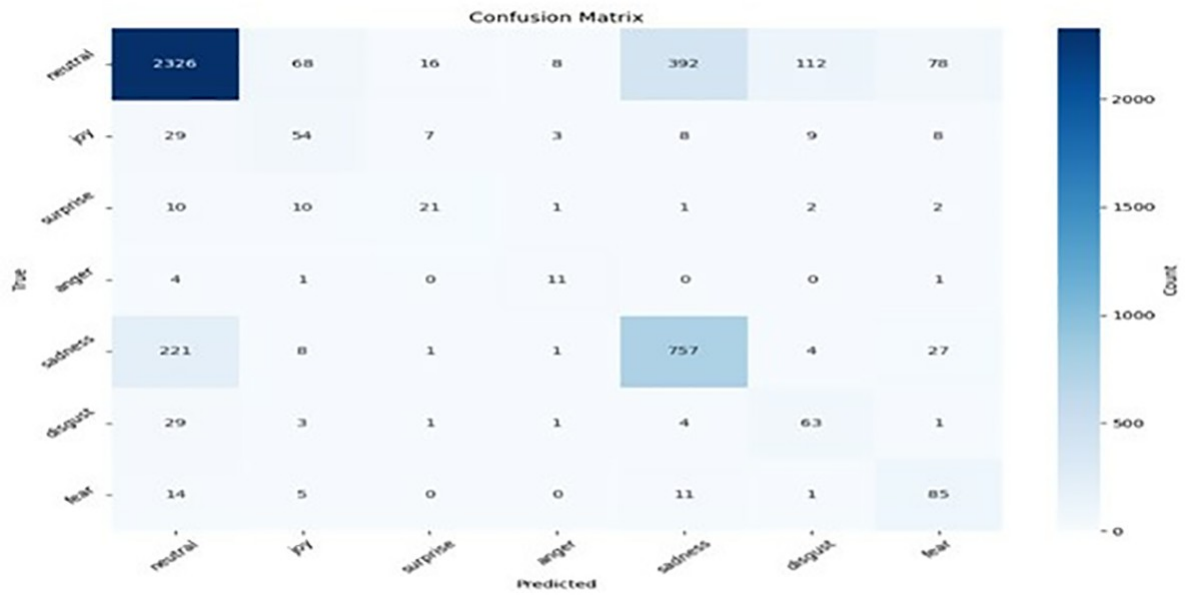
DailyDialog confusion matrix.

**Fig 10:**
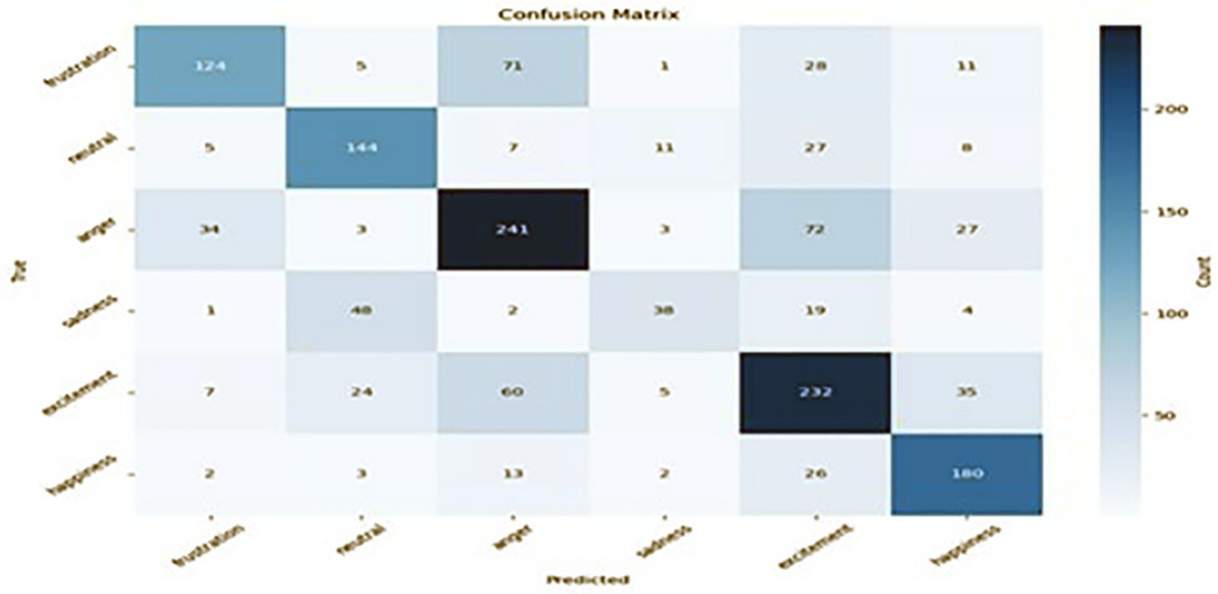
IEMOCAP confusion matrix.

We conducted a comprehensive hyperparameter tuning investigation to determine the effects of how various hyperparameters influence the model’s performance. Our findings revealed that the optimal combinations of optimiser, learning rate, and batch size significantly improve the model’s accuracy in emotion recognition. After optimally tuning the hyperparameters, we found that the Adam optimiser with a learning rate of 2e-5 and a batch size of 64 produces the optimal performance. Figs 7–10 result of the confusion matrix integrated MobileBERT model with Focal Weighted Loss and adversarial training achieves the best overall performance across MELD, EmoryNLP, DailyDialog, and IEMOCAP text emotion recognition datasets.

## 6 Conclusion and limitations

In this paper, we have proposed a novel loss function, Focal Weighted Loss (FWL), designed to address class imbalance in text emotion recognition tasks. By combining with adversarial training on low resources, we demonstrate that the compact MobileBERT model achieves superior performance on imbalanced datasets and is less sensitive to training the batch size, so it reduces the requirement of more computing resources. We have conducted our experiments on four widely used benchmark imbalanced text datasets, MELD, EmoryNLP, DailyDialog, and IEMOCAP. Our proposed method outperforms and performs well compared to large language models, demonstrating superior efficiency and achieving excellent results without requiring extensive computational resources. making our methods feasible for real-world applications where computational resources are limited.

### 6.1 Limitations

Our method has limitations. Our model not perform well on minority classes, which can limit its effectiveness in scenarios where emotions are imbalanced or have varying intensities. We need to conduct further research to compare our approach with other low-resource models and evaluate its performance on an imbalanced dataset. We need to explore the extension of our model to handle multi-modal inputs, which could provide a more comprehensive understanding of emotions.
